# Two Novel Dimorphism-Related Virulence Factors of *Zymoseptoria tritici* Identified Using *Agrobacterium*-Mediated Insertional Mutagenesis

**DOI:** 10.3390/ijms23010400

**Published:** 2021-12-30

**Authors:** Alexander Yemelin, Annamaria Brauchler, Stefan Jacob, Andrew J. Foster, Julian Laufer, Larissa Heck, Luis Antelo, Karsten Andresen, Eckhard Thines

**Affiliations:** 1 Institute of Biotechnology and Drug Research gGmbH, Hanns-Dieter-Hüsch-Weg 17, 55128 Mainz, Germany; jacob@ibwf.de (S.J.); thines@uni-mainz.de (E.T.); 2 Institut für Mikrobiologie und Weinforschung, Johann-Joachim-Becherweg 15, 55128 Mainz, Germany; brauchler@uni-mainz.de; 3 Institut für Biotechnologie und Wirkstoff-Forschung gGmbH (IBWF), Erwin-Schrödinger-Street 56, 67663 Kaiserslautern, Germany; Andy.Foster@sainsbury-laboratory.ac.uk (A.J.F.); Julian-Laufer@gmx.de (J.L.); 4 Microbiology and Wine Research at the Institute of Molecular Physiology (IMP), University of Mainz, 55128 Mainz, Germany; lheck02@uni-mainz.de (L.H.); luantelo@uni-mainz.de (L.A.); andresen@uni-mainz.de (K.A.)

**Keywords:** dimorphic switch, fungal dimorphism, RNA-Seq, transcriptomic analysis, forward genetics, reverse genetics, *Zymoseptoria tritici*, pseudohyphal growth, melanin, mycelium

## Abstract

Diseases caused by dimorphic phytopathogenic and systemic dimorphic fungi have markedly increased in prevalence in the last decades, and understanding the morphogenic transition to the virulent state might yield novel means of controlling dimorphic fungi. The dimorphic fungus *Z. tritici* causes significant economic impact on wheat production, and yet the regulation of the dimorphic switch, a key first step in successful plant colonization, is still largely unexplored in this fungus. The fungus is amenable to suppression by fungicides at this switch point, and the identification of the factors controlling the dimorphic switch provides a potential source of novel targets to control *Septoria* *tritici* blotch (STB). Inhibition of the dimorphic switch can potentially prevent penetration and avoid any damage to the host plant. The aim of the current work was to unveil genetic determinants of the dimorphic transition in *Z. tritici* by using a forward genetics strategy. Using this approach, we unveiled two novel factors involved in the switch to the pathogenic state and used reverse genetics and complementation to confirm the role of the novel virulence factors and further gained insight into the role of these genes, using transcriptome analysis via RNA-Seq. The transcriptomes generated potentially contain key determinants of the dimorphic transition.

## 1. Introduction

*Zymoseptoria tritici* (formerly *Mycosphaerella graminicola*) is a globally distributed plant-pathogenic fungus causing *Septoria* blotch disease, which is one of the most devastating diseases of wheat. The disease accounts for an approximately €1 billion per year expenditure in fungicides directed toward its control [1]. In spite of the commercial significance of the disease, the molecular basis of pathogenic development and plant colonization are still largely unexplored. Just a handful of genes have been demonstrated to encode virulence factors so far [2,3], and, largely, these genes were isolated individually, using reverse genetics, often based on their proven role in virulence in other phytopathogenic fungi. There is, therefore, a pressing need to employ next-generation sequencing (NGS) technologies, proteomics and metabolomics required to molecularly dissect the stages of the disease cycle in *Zymoseptoria tritici*, which is likely to have features unique to dimorphic fungi and not readily discovered by looking to pathogenicity factors present in strictly filamentous species. These insights have great potential to direct novel strategies for disease control. Indeed, such studies exploiting “omics”-technologies are beginning to provide a plethora of molecular mechanisms governing the pathogenic development of the fungus [4,5,6,7]. One key feature of the fungus is its ability to undergo the yeast-to-hyphae transition upon nitrogen deprivation and other environmental conditions. This change in growth forms—that occurs in both directions, dependent on the nutrient status and environmental factors—is referred to in the literature as a dimorphic switch [8,9,10]. Previous studies showed that dimorphism, and specifically the transition to the filamentous form, represents an essential prerequisite for infection, and this feature is shared with some other fungi, including other phytopathogenic fungi, such as *Ustilago maydis* or *Colletotrichum graminicola*; or systemic dimorphic fungi, such as *Candida albicans*, *Penicillium marneffei* or *Histoplasma capsulatum*, which are considerable threats for animals and humans [8,9,10]. *Z. tritici*’s dimorphic switch can be mimicked outside of the plant by transfer of the fungus from a yeast-like growth in nutrient-rich medium to a filamentous growth in a nitrogen-depleted medium [11,12]. To date, most of the genetic factors implicated in the dimorphic switch are signaling elements, including MAP kinases, cAMP pathway factors and G-proteins. Several additional factors implicated in the switch to the pathogenic form have been isolated by using reverse genetics in *Z. tritici* [3]. For example, deletion of the cyclin-encoding gene *ZtMCC* (previously published as *MgMCC*), orthologous to *Fusarium verticillioides FCC1*, results in delayed filamentation, atypical hyphal swellings, enhanced melanin formation, tolerance of oxidative stress and reduced virulence [13]. Inactivation of *Z. tritici MgMVE1*, which encodes a component of the Velvet regulatory complex, results in pleiotropic phenotypes, which include deficiencies in yeast–mycelial transition and changed responses to light on the switch [14,15], but no effect on virulence. *ZtALG2* (previously published as *MgALG2*) of *Z. tritici* was implicated in glycosylation of secreted proteins, and *ZtALG2* inactivation leads to a strain deficient in the dimorphic switch and impaired in secretion and completely abolishes wheat infection [16]. *ZtWor1*, *ZtVf1* and *ZtRlm1* encode transcription factors of *Z. tritici*, and deletion of these genes affects diverse developmental processes, including differentiation, asexual fructification, pathogenicity and, more specifically, hyphal branching and/or dimorphic switch [17,18,19]. A recent study examined the gene *Zt107320*, a homologue of the *Magnaporthe oryzae* transcription factor encoding the gene *MoCOD1*, and showed that its product regulates the dimorphic switch, as well growth rate and cell-wall composition in *Z. tritici*. Furthermore, deletion of the gene results in strains with reduced virulence during infection of wheat [20]. Finally, another study aimed at understanding the biological roles of different morphotypes in *Z. tritici* and at dissecting the associated transcriptional responses to tolerance of different environmental stresses showed that morphogenesis and the expression of virulence factors are co-regulated in *Z. tritici* [21].

In our previous report, we described a forward genetics approach for *Z. tritici*, complemented by a screening method that enabled us to identify dimorphic-switch-deficient mutant strains. This screen resulted in eleven interesting random mutants being impaired in the dimorphic switch [22]. Two of those mutants were previously characterized by using a genome walking approach. The corresponding genes were found to encode homologs of Ssk1p, representing a constituent of the HOG-pathway and Ade5,7p, involved in de novo purine biosynthesis, as previously characterized in other fungi. In the current study, we chose two further random mutant strains in order to characterize the corresponding genes functionally. Moreover, these mutants served as promising candidates for transcriptome analysis, since both exhibited altered morphology and physiological behavior compared to the wild type, being at the same time impaired in dimorphic transition. Together with wild-type IPO323 and the *ZtHOG1* mutant as reference strains, we determined the transcriptional profiles upon “dimorphic switch” –inducing conditions in order to gain insight into biological roles of the target genes in *Z. tritici*.

## 2. Results

We previously employed *Agrobacterium tumefaciens* mediated transformation (ATMT) to undertake insertional mutagenesis to isolate dimorphic switch-deficient mutants for *Zymoseptoria tritici* from a collection of 10,000 transformants [22]. Here, we were focused on detailed characterization of two genes of the putative set of dimorphism-related genes previously presented and assigned to the random mutant strains *myco#5* and *myco#56*. Both mutants exhibit interesting phenotypic features. The mutant strain *myco#5* was found to be associated with the enhanced production of mycelium under different growth conditions, encompassing both complete and N-deprivation media. Interestingly, the hyperfilamentation phenotype was also observed on YEG complex medium, which normally induces yeast-like growth in wild-type strains. In contrast, the phenotype of the *myco#56* mutant is associated with a pronounced reduction of mycelium formation, while displaying an aberrant phenotype which resembles the pseudohyphal growth of the culture. Interestingly, both mutant strains were markedly reduced in their virulence toward the wheat cultivar Riband, since no typical disease symptoms were apparent.

### 2.1. Identification of T-DNA Insertion Sites in myco#5 and myco#56 Mutant Strains and Gene Sequence Analysis

**Figure 1:**
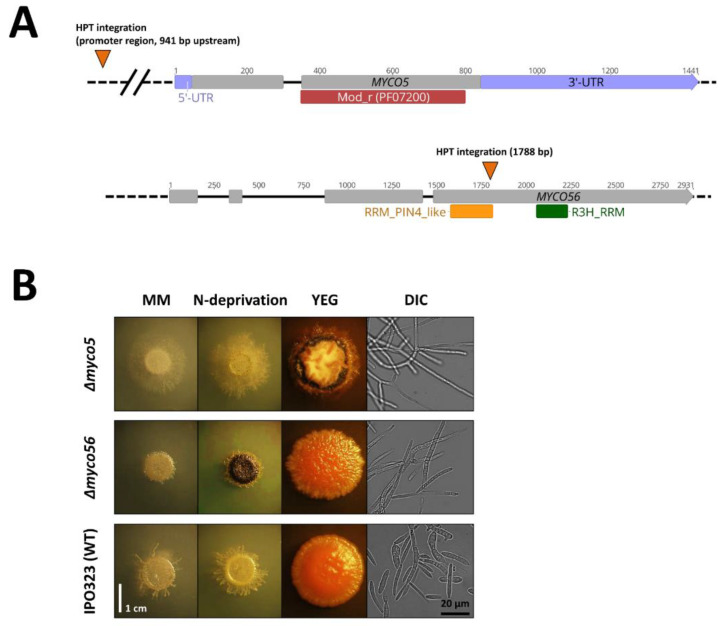
(**A**) Structure of the predicted target genes *MYCO5* and *MYCO56* and the position of the inserts in the mutants. Sites of T-DNA integration (*HPT*) in the genes are indicated. The numbers indicate the length of the annotated genes. The highlighted bars show the functional domains present in the predicted gene products and found by using InterProScan. Black lines indicate intron, and gray bars indicate exons. (**B**) Phenotypes of the mutants are shown by macroscopic analysis of colony morphology after 21 days’ growth on minimal medium (MM), N-deprivation medium and YEG medium. The micrographs (DIC) indicate the spore morphology following 3 days’ growth in YEG liquid medium.

A genome walking approach was used to identify the gene loci potentially disrupted by insertion events, as previously described [22,23]. In the *myco#5* random mutant, T-DNA integration occurred at position 4.409.450 on chromosome 1, 941 bp 5′ of the annotated translational START site of the predicted gene ID 66947 (*MYCGR3_66947*; GenBank Accession ID: XP_003857106.1) (Figure 1A). *MYCO5* is predicted to encode a protein related to *Saccharomyces cerevisiae* Vps37, a mod_r (PFAM: PF07200) domain-containing protein (Figure 1A). The similarity is largely confined to the C-terminus of the proteins at the site of the mod_r domain. Together with Vps23, Vps28 and Mvb12, Vps37 forms the ESCRT-I complex, which mediates endosomal sorting at the multivesicular body (MVB) [24]. Potential homologues of the predicted gene product were identified in related species, such as *Z. brevis* and *Z. arabidae*, and also in more distant phytopathogenic filamentous fungi harboring appressorial and non-appressorial modes of host penetration.

In the *myco#56* random mutant, the T-DNA integration was localized to chromosome 8 at position 1.351.161, 1788 bp downstream of the start codon in the ORF of the gene with JGI Protein ID 110503 (*MYCGR3_110503*; GenBank Accession ID: XP_003850341.1) (Figure 1A). BlastP searches showed that the closest related hitherto investigated protein to Myco56p present in the NCBI non-redundant protein (nr)-database was Cip2p of *Schizosaccharomyces pombe* (Accession No. NP_592895.1; E-value: 3 × 10^−41^), with 32% amino acids identity. Myco56p is predicted to be 743 amino acids in length, with an RRM_6 domain (PF14259) between amino acids 295 and 365 and an R3H domain (PF01424) present at 469–510 within the protein. RRM domains have been found to be a feature in proteins participating in RNA processing [25]. One example of RRM-domain-type proteins is the Csx1p-, Cip1p- and Cip2p-containing complex that was previously demonstrated to regulate the response to oxidative stress in *S. pombe* by influencing RNA decay [26,27]. Cip2p additionally has a R3H motif thought to play a role in sequence-specific binding to single-stranded nucleic acids [28]. BLASTp searches indicate that Myco56p similarity to its homologues occurs predominantly across the RRM and R3H domains.

To establish whether *MYCO5* and *MYCO56* are conserved across a range of fungal species, a phylogenetic analysis was carried out. Both filamentous and systemic dimorphic fungi, as well as closely and distantly related species, were considered, and the resultant orthologous sequences were used to make phylogenetic trees. We additionally included an orthologous sequence of *MYCO5* in the closely related species *Z. pseudotritici*. *Mycgr3_103744* and *Mycgr3_73484* are non-homologous, and they are the next most similar sequences found by using BLASTp and were included as an outgroup. As anticipated, this analysis showed that the orthologues in the *Capnodiales* family were grouped together in one clade, indicating their origin from a sole progenitor (Supplemetary Materials Figure S1). In the case of *MYCO5*, no orthologs were found in *Candida albicans*, *Ustilago maydis*, *Cryptococcus neoformans* and *Saccharomyces cerevisiae*, thus indicating a possible loss of genetic information in these species due to evolutionary adaptation.

### 2.2. Targeted Gene Deletion Corroborates the Role in the Dimorphic Switch and Virulence for the MYCO5 and MYCO56 Genes

**Figure 2:**
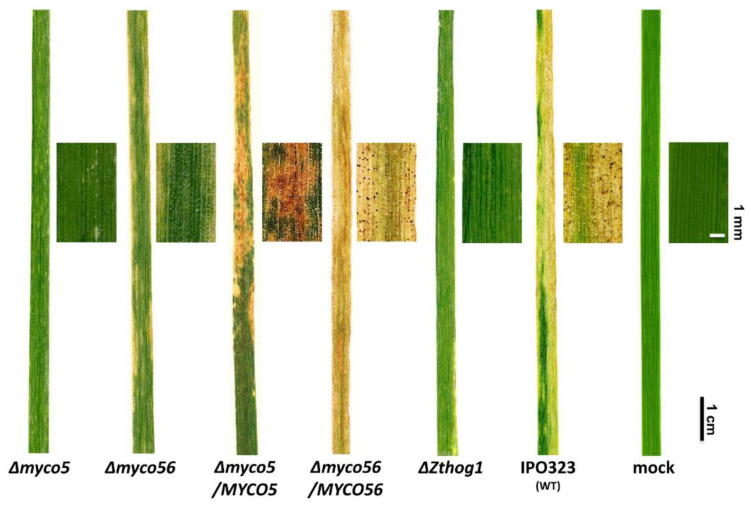
Virulence of *Z. tritici* Δ*myco5* and Δ*myco56* strains. The effects of the gene deletion on disease development in the susceptible wheat cv. Riband after 21 days after inoculation (dpi) are indicated. The wild-type IPO323 and Δ*Zthog1* are used as positive and attenuated virulence controls, while “mock” is a negative control (inoculated with water only). Infections were carried out at 22 °C with 80% humidity and a 16 h/8 h light cycle. The strain Δ*myco5* proved to be non-pathogenic, while Δ*myco56* strains, such as Δ*Zthog1*, are severely reduced in virulence. Successful infection was observed for IPO323, which formed mature pycnidia at 21 dpi. Full virulence was completely restored for the complementation strains Δ*myco5*/*MYCO5* and Δ*myco56*/*MYCO56* generated by reintroduction of the full-length genes in the respective mutants.

Gene deletion of *MYCO5* and *MYCO56* in the wild type of strain IPO323 was used to confirm the association of observed phenotypes and T-DNA insertions in the mutant strains. Southern Blot analysis was used to identify strains where the respective gene had been deleted by homologous recombination of the gene deletion cassettes (Supplementary Materials Figures S2 and S3). The resultant gene deleted mutants confirmed the role of *MYCO5* and *MYCO56* in the dimorphic switch and virulence (Figure 1B). The reduced virulence to susceptible wheat cultivar Riband was confirmed in every confirmed gene deleted strain tested (Figure 2). The Δ*myco5* strain was shown to be non-pathogenic, while the Δ*myco56* strain had a markedly reduced virulence compared to IPO323. Even though the Δ*myco5* mutant predominantly grows in a filamentous form associated with virulence, it caused no visible signs of disease on wheat plants (Figure 2). The Δ*myco56* strain, meanwhile, caused very occasional necrotic lesions and drastically reduced numbers of pycnidia after the latent period of infection. The wild-type strain IPO323 and the complemented mutants generated by reintegration of intact copy of the respective genes were indistinguishable in their ability to cause disease of wheat.

### 2.3. Transcriptome Analysis of Selected Strains during the Dimorphic Transition on Artificial Medium

To probe the role of the target genes on the yeast-to-hyphal transition, we generated the transcriptomes of the Δ*myco5* and Δ*myco56* strains by using RNA-Seq at the time of switching. The wild-type stain IPO323 and Δ*Zthog1* were used as controls. Strains were incubated on cellophane-coated N-deprivation agar plates for 7 days, at 18 °C, prior to RNA isolation. Analysis of the distribution of reads aligned to the reference genome suggested a sufficient sequencing depth in all the strains examined (Supplementary Materials Figure S4A,B).

**Figure 3:**
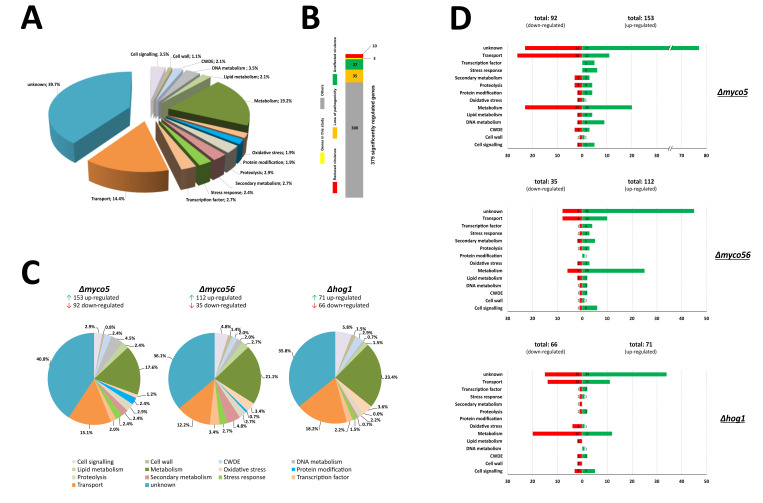
Transcriptome analysis of the strains IPO323, Δ*myco5*, Δ*myco56* and Δ*Zthog1* grown under the dimorphic switch inducing condition. (**A**) Pie chart showing the custom categories of the products of all the DE genes (375) obtained from RNA-Seq analysis that were categorized by using data obtained from JGI server and Blast2GO analysis. Significant differential expression was defined as average expression altered by a factor >2.5× at one instance of pairwise comparisons across all the strains investigated and with a q-value threshold of less than or equal to 0.05. (**B**) Identification of virulence genes. Searches using the predicted amino acid sequences from the set of DE genes was undertaken by using BLASTp (E-value cutoff of ≤10^−30^) against the PHI-Base database (version 4.1) to assess potential involvement in host–pathogen interaction. (**C**) Pie charts showing the biological categories of all the DE genes obtained from the RNA-Seq studies. The pie chart shows the percentage of the genes expressed for each biological process. Biological categories were assigned manually based on the presence of conserved functional domains, GO annotation and/or the best meaningful match, using BLASTp. Genes identified by using Cuffdiff-analysis were filtered for an absolute fold-change value ≥2.5 and q-value threshold of 0.05. (**D**) Functional groupings of up- and downregulated DE gene products. Bar charts show a functional classification based on the manual annotation of the DE genes for each strain-specific transcriptome. Red bars represent functional categories for products of the genes with reduced transcript abundance, while the green bars represent those for those with increased transcript abundance. The total number of down- and upregulated genes for each biological category is also indicated.

In total, 375 significantly differentially regulated transcripts were identified. Genes were deemed differentially expressed by using a cutoff where the average expression was found to be affected by factor of at least 2.5-fold in one instance of pairwise comparisons across all the strains investigated and that the q-value (representing the false discovery rate of the *p*-value) was less than or equal to 0.05. Classification of genes by to their predicted biological functions gave 14 custom functional groups, which were named in accordance with annotated GO and KOG (euKaryotic Orthologous Groups) JGI data, in addition to the combined results retrieved from BLASTp and InterProScan analysis. Blast2GO analysis was employed to reassess and update the GO data from the Joint Genome Institute. Consequently, BLASTp searches of the NCBI “nr”-database, together with GO analysis, led to the identification of 226 genes (60.3%) that could be classified into functional categories (Figure 3A). The most enriched category with approximately 19% of the significantly differentially expressed genes was represented by metabolism-related proteins, followed by “transport” with near to 14% of annotated genes. The other 149 genes (39.7%) were predicted to code for hypothetical products and were classified as “unknown”, because no GO annotation terms were uncovered (Figure 3A). Additionally, using a custom database of known virulence factors (applied as PostgreSQL Database), the majority of which were taken from the PHI-Base database version 4.1, we could pinpoint a set of orthologous genes, whose role in the pathogenicity-related processes has been already demonstrated in other pathogenic fungal species. Assignment of these pathogenicity-related genes used three criteria: “loss of pathogenicity”, “reduced virulence” and “hypervirulence” or “unaffected virulence”. Henceforth, 10 genes were identified, whose disruption or deletion in other pathogenic fungi leads to “loss of pathogenicity” (Figure 3B). Furthermore, there were 35 genes whose disruption or deletion leads to a reduced virulence in other pathogenic fungi. Most of these genes code for transporters (mainly MDR transporters) and/or are involved in the vague functional class “metabolic processes”. Representatives of this set are, for example, *PTH11* from *Magnaporthe oryzae* [29], *MEP1* from *Coccidioides posadasii* [30] and *SCD1* from *Colletotrichum lagenarium* [31]. Additionally, 27 differentially regulated genes had homologues in other pathogenic fungal species, where they are linked to hypervirulence or found to be unaffected in virulence according to the PHI-Base database (Figure 3B). Relatedly, again, many of these genes code for transporters or proteins potentially involved in metabolic processes.

To understand the transcriptional effects of perturbation of *MYCO5*, *MYCO56* and *ZtHOG1*, we determined which strain-specific genes display significantly altered transcript abundance in the respective mutant compared to IPO323 (henceforth, these genes are referred to as DE genes). Similarly, genes were only considered DE if they exhibited a fold change value of at least 2.5, together with a false discovery rate *p*-value of ≥0.05. For exploratory purposes, we employed a previously used interactive scatterplot visualization tool that was created by using the Python/Bokeh framework accessible through: https://mybinder.org/v2/gh/Alexyem1/Myco_2021.git/main?urlpath=%2Fproxy%2F5006%2Fbokeh_app (last accessed on 1 December 2021) [32]. The target gene-specific transcriptomes based on RNA-Seq analysis follow.

Furthermore, for each of the mutant strains examined, we listed the top twenty ranked genes with a very comparable transcription profile across all strains (Supplementary Materials Figure S5). In addition, RNA-Seq data were compared to publicly available datasets from studies aimed at examining expression profiles in planta. With this approach, we attempted to find DE genes, whose products may be important for pathogenicity or may exert their functions in plant–pathogen interaction. These studies encompass the previously published RNA-Seq analysis [4,5,6,7], as mentioned earlier, which was particularly directed to the identification of DE genes following the complete infection cycle (at distinct time points) of *Z. tritici* in susceptible host plants. Hence, by focusing primarily on DE genes commonly shared across these datasets, we provide a list of potential candidates putatively involved in the critical steps of pathogenic development of the fungus (Supplementary Materials Table S1).

#### 2.3.1. Genome-Wide Transcriptional Effects of MYCO5 Perturbation

The RNA-Seq analysis showed that there were 245 DE genes from the Δ*myco5*:IPO323 comparison; this was composed of 153 genes upregulated and 92 downregulated in Δ*myco5* (Figure 3C,D). These 245 DE genes were markedly enriched in proteins implicated in metabolism (17.6%) and transport-related processes (15.1%). A comparable observation was seen when individually examining up- and downregulated genes where the predicted products of the most abundant transcripts were found to be associated with metabolism-related pathways or in transport-related processes (Figure 3D). Notable within the predicted transporter-encoding genes exhibiting reduced transcript abundance were genes predicted to encode transporters potentially mediating the uptake of extracellular glucose or other monosaccharides. Further notable groups of DE genes were predicted to encode (lyso)phospholipases, esterases and triacylglycerol hydrolases. This led us to speculate that perhaps an altered lipid profile of Δ*myco5* may explain its aberrant growth behavior. It was striking too that, only in the *MYCO5* deleted mutant, a gene predicted to encode a FabD/lysophospholipase-like protein (*Zt109795*) and two genes which could code for hypothetical triacylglycerol hydrolases (*Zt81448*, *Zt105080*) exhibited highly elevated transcript abundance. Furthermore, three genes predicted to encode a sterol esterase (*Zt29873*), a lipase (*Zt74078*) and fatty-acyl-CoA synthase (*Zt39405*) showed decreased transcript abundance only in the Δ*myco5* mutant. A set of genes predicted to encode (chloro)peroxidases and Cu/Zn-dismutases were found to be downregulated in the Δ*myco5* mutant, suggesting a reduced ability to detoxify hydrogen peroxide. It is additionally notable that, in the wild-type strain IPO323, these genes are transcribed throughout the course of infection (Supplementary Materials Figure S6). Interestingly, these genes have additionally been implicated in virulence on the basis of transcriptomic analyses of *Z. tritici* in other studies [6,7].

**Figure 4:**
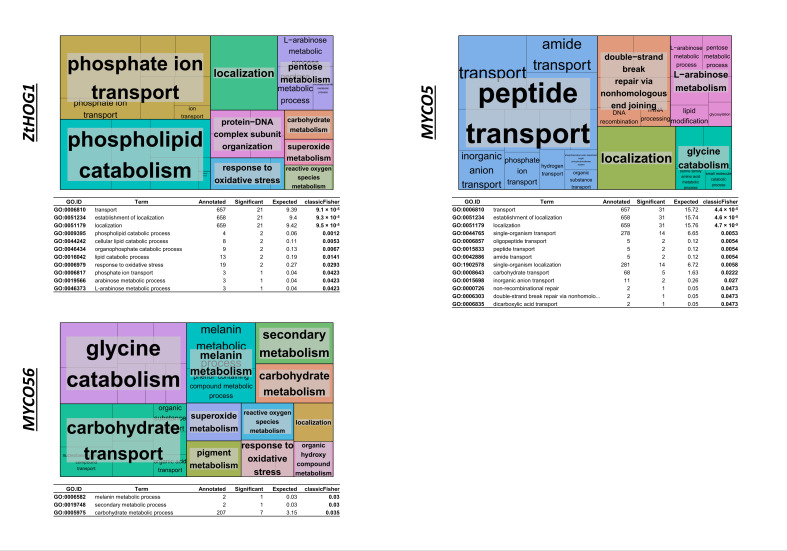
GO enrichment analysis of differentially expressed genes in Δ*Zthog1*, Δ*myco5* and Δ*myco56*. GO terms enriched in the DE genes for each of the mutant strains studied (*ZtHOG1*, *MYCO5* and *MYCO56*) were analyzed with the aid of the TopGO package in R, and the first fifty GO clusters were processed by using the web-based tool REVIGO. GO terms’ redundancy was subtracted, and the GO terms for biological processes were clustered in TreeMap plots, where each rectangle represents a single cluster. Similar colors of the rectangles indicate semantic similarity. The magnitude of the rectangles reflects the *p*-value obtained from TopGO analysis (the larger the size, the smaller the *p*-value and the greater the enrichment factor for respective GO term). For the TreeMaps, the adjoining table shows those GO clusters identified by using TopGO evaluation and which were significantly enriched according to classic Fisher analysis with a *p*-value ≤ 0.05.

By employing GO-enrichment analysis DE genes were clustered by focusing chiefly on “biological processes”. To gain the greatest value from the processed data, fifty of most enriched gene clusters were calculated (Figure 4). Clustering of the GO terms enriched in the mutant perturbed for *MYCO5* showed that many of DE genes were related to “transport” and included transport of carbohydrate (GO:0008643), peptides (GO:0015833) and inorganic ions (GO:0015698). Further enriched GO terms in the Δ*myco5* strain were related to lipid metabolism (GO:0030258, GO:0030259) and catabolic processes (GO:0008643, GO:0019566, GO:0046373).

In addition, a number of hypha-specific gene homologs were apparent in the set of DE genes, previously characterized as virulence factors in other fungi, e.g., *TEC1* (*Zt92404*) and *LIG4* (*Zt68344*) from *Candida albicans* [33,34,35] and *GEL2* (*Zt102341*) from *Aspergillus fumigatus* [36], as well as *MEP1* (*Zt43998*) from *Coccidioides posadasii* encoding a metalloproteinase, which is secreted during endospore differentiation [30]. Moreover, the *MYCO5* transcriptome analysis showed a significantly increased transcript abundance for a gene predicted by homology to code for a t-SNARE protein (*Zt58641*), suggesting a potential role for this product in cell polarity, exocytosis and apical growth, as demonstrated for similar proteins [37]. We also found DE genes whose products are predicted to be involved in cell wall biogenesis and remodeling, including a chitinase, a 1,3-β-glucanase, several glycosyl transferases and one hydrolase.

#### 2.3.2. Genome-Wide Transcriptional Effects of MYCO56 Perturbation

While dissecting the transcript changes present in the mutant Δ*myco56*, 112 genes were shown to be upregulated and 35 genes were downregulated during the “dimorphic switch”–inducing condition (Figure 3D). The genes with increased transcript abundance and with functional annotation were enriched in biological processes, such as “metabolism”, as well as processes related to transport activity, “cell signaling” and “secondary metabolism”. Most of the downregulated transcripts were enriched in “metabolism” and “transport” (Figure 3C,D). In particular, we identified five core genes (*Zt96592*, *Zt68710*, *Zt18775*, *Zt87994* and *Zt87993*) of melanin biosynthesis which were highly expressed compared to the wild type, Δ*Zthog1* and Δ*myco5*. These genes are predicted to participate in melanin biosynthesis and represent the likely homologues of *Colletotrichum lagenarium PKS1*, *BRN2* and *SCD1* which code for the polyketide synthase, the T4HN reductase and the scytalone dehydratase respectively [31,38,39], as well as the T3HN-reductase-encoding genes (*BRN1* or *THR1*) [40] and *CMR1,* which codes for a transcription factor that regulates the transcription of the other genes in the melanin biosynthesis cluster [41]. These findings were additionally supported by GO clustering analysis of the differentially expressed transcripts with the aid of REVIGO, which showed enrichment of the melanin biosynthesis class of genes (GO:0006582), as well as secondary metabolic process (GO:0019748), amino acid catabolic processes (GO:0006546 and GO:0009071), carbohydrate metabolic process (GO:0005975) and carbohydrate transport (GO:0008643) groupings of genes (Figure 4). Additionally, enrichment of genes involved in oxidative stress response (GO:0006801, GO:0006979) was demonstrated in Δ*myco56* compared to IPO323. Interestingly, the transcriptome profile in the *MYCO56* deleted strain also revealed a significant increase in transcript abundance for the *FLBD* homolog, whose product plays a critical role in conidiation and conidiophore development in a number of fungi, including *M. oryzae* [42].

#### 2.3.3. Genome-Wide Transcriptional Effects of ZtHOG1 Perturbation

Our analysis of the Δ*Zthog1* transcriptome revealed 137 DE genes: 71 with an increased transcription and 66 genes with a decreased transcription. The most notable enriched groupings were “metabolism”, “transport” and “cell signaling”, containing 23.4%, 18.2% and 5.8% of DE genes, respectively. The genes classified in the category “oxidative stress response” were also enriched Δ*Zthog1* transcriptome. Many of the genes with enhanced expression in the mutant were predicted to code for proteins participating in metabolism, transport related processes and cell signaling. The majority of the downregulated genes are grouped in the categories of processes related to “metabolism”, “transport” and “oxidative stress”. This finding was supported by REVIGO clustering (Figure 4). Within the enriched clusters we noted metabolism (GO:0008152), oxidative stress response (GO:0004601 and GO:0006118) and transport-associated processes (GO:0006810), including numerous ABC- and MDR-transporters. Additionally, the clusters carbohydrate catabolic process (GO:0019321, GO:0046373 and GO:0019321) and lipid metabolic/catabolic process (GO:0016042, GO:0044242 and GO:0006644) were significantly enriched. Notably, genes which potentially code for detoxification enzymes, peroxidases, ABC transporters and UDP-glucosyl/glucuronosyl transferases showed decreased transcription. In addition, a number of gene homologs were apparent in the set of DE genes, previously characterized as virulence factors in other fungi, e.g., *PLB1* (*Zt107391*) from *Candida albicans* [43] and *PTH11* (*Zt111657*) from *M. oryzae* [29]. Moreover, we found that the transcriptomes of both Δ*Zthog1* and Δ*myco56* mutants had a marked overlap, particularly among genes whose products are predicted to have a role in cell signaling, cell-wall remodeling and (oxidative) stress response. This overlap was supported by PCA analysis (Supplementary Materials Figure S7).

### 2.4. There Are Genes Unique to Zymoseptoria tritici among the DE Datasets

**Figure 5:**
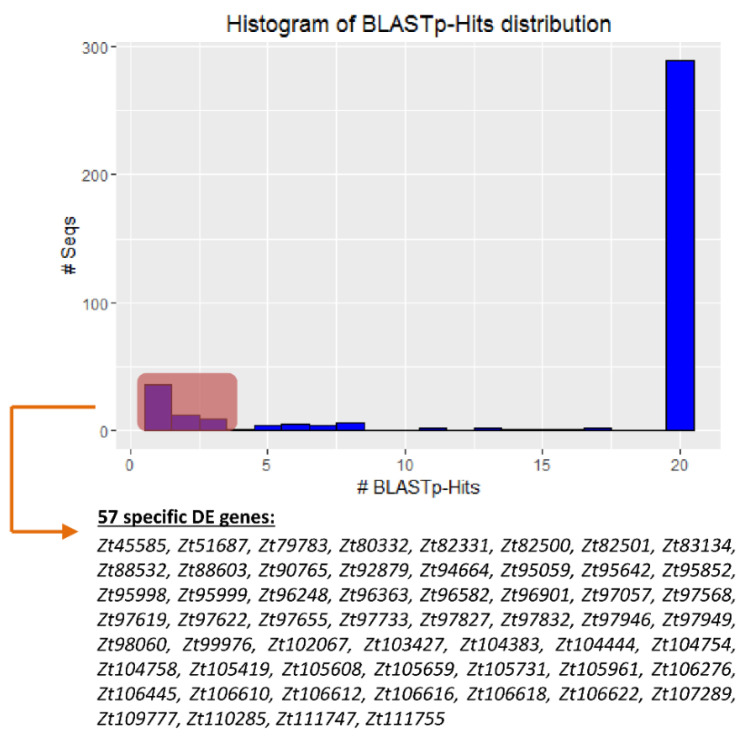
Histogram indicating the distribution of the BLASTp hits of DE genes. The predicted amino acid sequences of the 375 DE genes from the RNA-Seq analysis were searched by using the NCBI “nr”-database to identify potential homologs. Gene products with just 1, 2 or 3 orthologues in other fungal species were considered as potentially “unique” or “*Z. tritici*-specific”, and thus represent attractive candidate genes for further study.

Probing only for significantly differentially expressed genes with no, or rare, homologs in other fungal genomes could represent a path to identify factors unique to the particular pathogenic development in *Z. tritici*. To find such genes, we undertook a comparison of the predicted sequences of the products of the DE genes we found in our RNA-Seq analysis. Searches were made against the NCBI fungi-database, using BLASTp. Among the 375 genes DE genes, we found that 280 had 20 or more orthologues across the different fungal species (Figure 5). Of these, 37 genes were found to have multiple (ranging from 4 to 19) potential orthologues in comparisons to the other fungal species. Additionally, 57 of the DE genes were shown potentially to be unique to *Z. tritici* or generally for *Zymoseptoria* phylum, based on currently available data, because they had just one, two or three orthologues in the other species (Figure 5). Among these “unique” genes, a vast majority were assigned to genes encoding hypothetical proteins (no functional annotation). Functional information in the JGI database was found for just two genes for which the corresponding gene products are a small threonine-rich protein (*Zt105419*) and flocculin mucin-like precursor (*Zt105608*).

### 2.5. Δmyco56 Has Increased Pigmentation Following Growth on a Nutrient-Depleted Medium and When Grown as a Submerged Culture, while Δmyco5 Exhibits Thermo-Sensitivity

**Figure 6:**
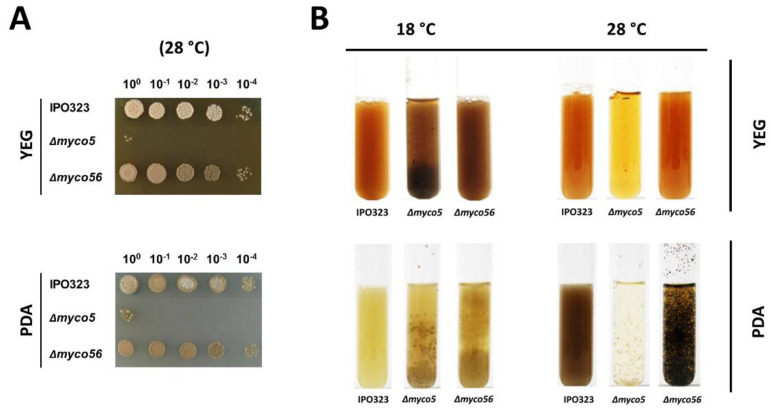
Growth of IPO323 and the mutant strains at elevated temperatures. (**A**) Axenic growth of *Zymoseptoria tritici* wild-type and generated mutant strains at increased temperature. YEG-grown cultures of the wild-type strain IPO323 and mutant strains Δ*myco5* and Δ*myco56* were spotted onto YEG/PDA plates as serial dilutions (1.5 μL; 5 × 10^7^ spores/mL). Cultures were imaged following 7 days’ growth at 28 °C. (**B**) Axenic cultures of *Z. tritici* strains showed unusual cell aggregates and clumpy growth in the Δ*myco5* strain in PDA or YEG and for the Δ*myco56* strain in PDA. Cultures were agitated prior to imaging. Cultures for each condition were routinely incubated in 100 mL flasks, without baffles, for one week, with shaking at 120 rpm. Before imaging, strains were transferred to glass tubes. For Δ*myco5*, a drastically reduced growth was apparent at elevated temperatures.

When growing the strains as submerged cultures, we found that Δ*myco5* grew in YEG and PDA at 18 °C predominantly in the filamentous form, and although it produced yeast-like conidia, these were very reduced in number. We noted that growth was drastically impaired when the strain was grown at 28 °C, both on a solid medium (Figure 6A) and in a liquid culture (Figure 6B), thus indicating heat sensitivity. Growth in liquid culture was aberrant in comparison to wild-type, with the formation of dense spheroid-like mycelial aggregates. This suggests that there may be a reduced capacity for nutrient uptake due to the decreased surface area.

Δ*myco56* has a pseudohyphal-like growth mode in submerged culture, similar to that observed on solid YEG medium. Δ*myco5* and Δ*myco56* both grow in YEG at 18 °C as melanized cultures. Notable was a highly increased pigmentation in Δ*myco56* in comparison to the wild type that was seen in both YEG and PDA liquid media, indicating potentially increased activity of genes involved in 1,8-dihydroxynaphtalene (DHN)-melanin biosynthesis. The increased melanin content of the Δ*myco56* was also seen on N-deprivation agar medium and water agar, but not on the minimal medium (Figure 1B). The altered pigmentation seen on water agar became apparent only after prolonged incubation (>21 days; data not shown), implying that utilization and trafficking of intercellular energy sources may lead to the generation of precursors required to melanize. Together, the RNA-Seq results and the phenotypic observations indicate that increased melanization in the mutant is not due solely to nutrient deprivation but could be related to *MYCO56*’s involvement in the regulation of the genes of melanin biosynthetic cluster.

### 2.6. Vegetative Growth and Stress Response of the Δmyco5, Δmyco56 and ΔZthog1 Mutants

The transcriptional reprogramming found under N-starvation response showed that many genes exhibit differential expression in a strain specific manner. To validate the RNA-Seq data and to further explore how the phenotypes of mutants used fit with the observed transcriptional changes, vegetative growth assays were carried out. Led by the transcriptional changes seen, we made cultures by using varied stressors (including oxidative stressors, transient metal stress and cell-wall stressors) and at two different temperatures, namely 18 and 28 °C, with the latter temperature being a heat stress for *Z. tritici*.

### 2.7. MYCO5 Plays a Role in the Oxidative Stress Response

**Figure 7:**
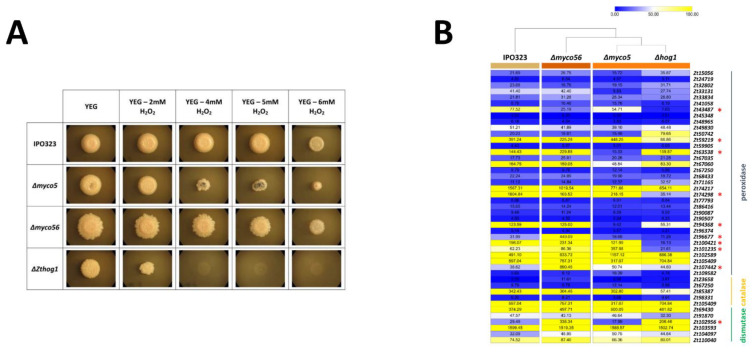
Susceptibility of mutant strains to oxidative stress. (**A**) Morphologies of colonies of *Z. tritici* IPO323 and the mutants generated in the current study under oxidative stress. Wild-type strain and mutant strains were spotted as 1.5 μL spots from a 5 × 10^7^ spores/mL spore suspension and grown on YEG agar medium, with the concentrations of H_2_O_2_ indicated. Plates were documented following 7 days’ growth at 18 °C. *ZtHOG1* is more susceptible to oxidative stress, while Δ*myco5* has a slightly reduced growth. (**B**) Transcription of the potential (chloro)peroxidases, catalase and superoxide-dismutase-encoding genes identified by using RNA-Seq data. Gene-expression data were clustered in one dimension by hierarchical agglomerative clustering with complete linkage. One minus Pearson correlation was used as the similarity metric for the *Z. tritici* strains. The detected expression profiles reveal, on average, a lower expression of listed genes in the case of Δ*myco5* and Δ*Zthog1*, while Δ*myco56* was the highest. Red asterisks (*) show DE transcripts with significantly changed transcription in at least two strains.

As indicated in Figure 7A, 6 mM H_2_O_2_ in YEG medium impeded the growth of all the strains; however, susceptibility did vary among the strains. The growth conditions allowed us to measure the radial diameter of colonies. Exposure to lower levels of H_2_O_2_ (2 mM) led to growth inhibition of Δ*Zthog1* and Δ*myco5* markedly greater than growth inhibition of the wild-type strain, with inhibition of Δ*Zthog1* being the most pronounced. Δ*Zthog1* had retarded growth on 2 mM H_2_O_2_ supplemented YEG medium but ceased growth totally upon exposure to 4 mM H_2_O_2_. In the case of the Δ*myco5* strain, strong inhibition of growth at 4–6 mM H_2_O_2_ compared the wild-type and Δ*myco56* strains was apparent (Figure 7A).

Complementation was used to prove that the observed defects could be rescued by reinstating a wild-type copy of the defective gene driven by native promoters in the respective strains. The phenotypes seen correlate with the transcription profiles obtained from RNA-Seq data, in that Δ*Zthog1* and Δ*myco5* showed on lower transcript abundance of genes whose products may participate in response to H_2_O_2_ and superoxide stress, including potential chloroperoxidases and superoxide dismutases (Figure 7B). Comparing the transcriptional profile of the Δ*myco5* mutant with that of IPO323, five genes (*Zt33131*, *Zt63538*, *Zt67060*, *Zt94368* and *Zt74298*) showed downregulation, while only two genes (*Zt101235* and *Zt102589*) shown increased expression. In contrast, Δ*myco56* had the highest number of peroxidase and dismutase encoding genes with significantly altered transcript abundance among all strains, such as *Zt96677*, *Zt107442* and *Zt102956*. For the other genes potentially involved in oxidative stress response, very similar transcriptional levels across all the strains examined were found.

### 2.8. MYCO5 Is Involved in the Detoxification of Transient Metals

**Figure 8:**
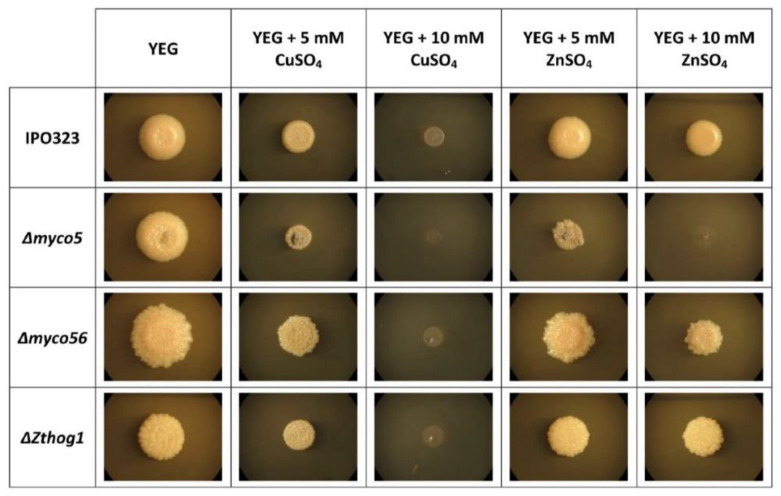
Colony morphology of *Zymoseptoria tritici* IPO323 and the mutants studied during transient metal stress. Δ*myco5* had increased sensitivity to elevated levels of metal ions. Strains were grown as YEG cultures and then spotted from a spore suspension (1.5 μL; 5 × 10^7^ spores/mL) to YEG solid medium supplemented with 5 mM CuSO_4_ and 10 mM ZnSO_4_. Plates were imaged following 7 days’ growth at 18 °C.

RNA-Seq data from the Δ*myco5* transcriptome showed that a group of the DE transcripts are predicted to code for ion transporters, indicating a likely role in the detoxification of different metal ions. The majority of these transcripts had reduced abundance in Δ*myco5*. To further confirm the susceptibility toward metals, the strains were incubated on YEG medium supplemented with sublethal concentrations of the transient metals, including CuSO_4_ and ZnSO_4_. Incubation of the Δ*myco5* strain supplemented with 5 mM CuSO_4_ YEG led to growth impairment (Figure 8). None of the strains examined grew at 10 mM CuSO_4_. Incubation of the strains on YEG with 5 mM ZnSO_4_ allowed growth that was similar for all the strains, except in the case of Δ*myco5* blocked at 10 mM ZnSO_4_, whereas IPO323 and the other mutants could tolerate this amount of ZnSO_4_. Complementation of the Δ*myco5* confirmed the link between the loss of the *MYCO5* gene and the observed phenotype, because the wild-type response to zinc was fully restored by reinstating the *MYCO5* gene, using its own promoter and terminator sequences (data not shown).

### 2.9. MYCO5 Has a Role in Maintaining Cellular Lipid Status

**Figure 9:**
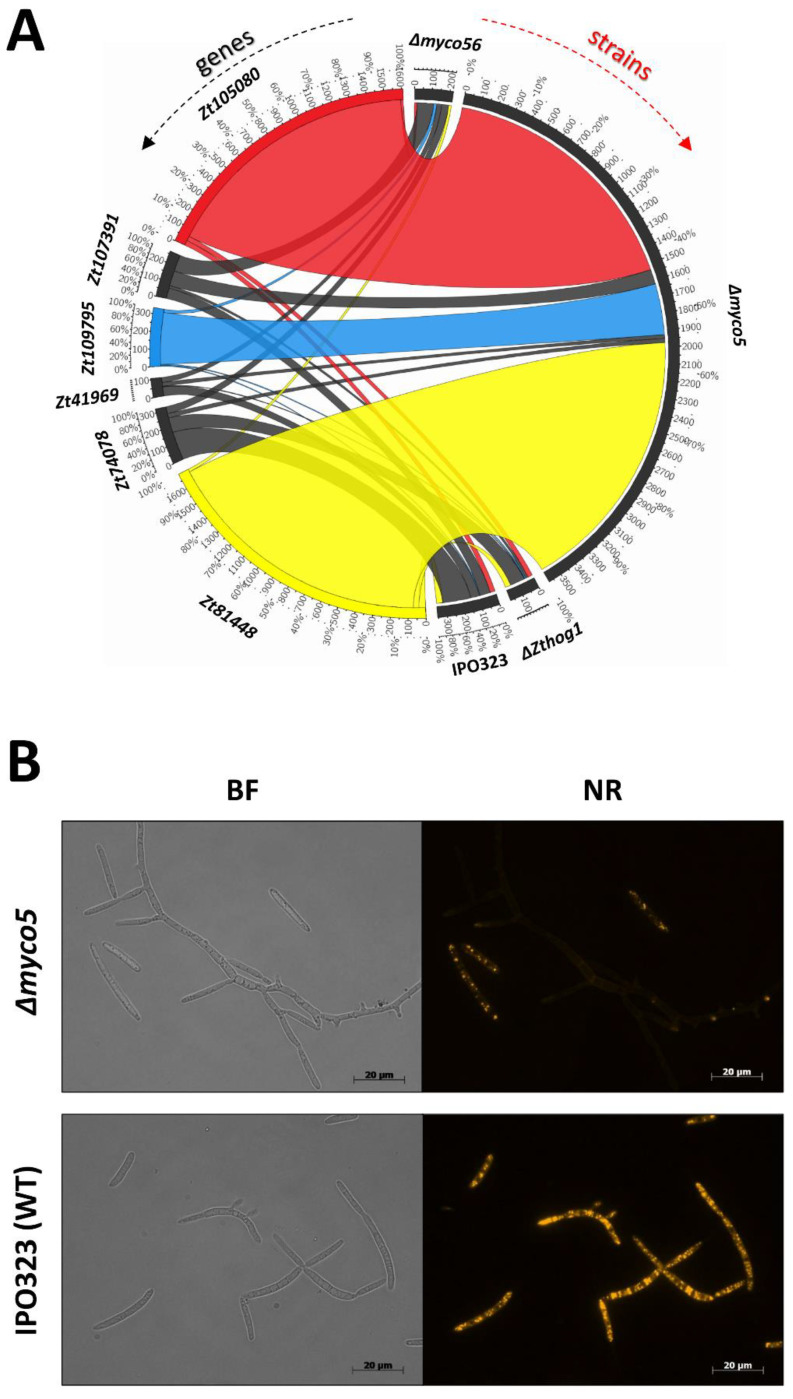
Aberrant lipid metabolism in the strain Δ*myco5*. (**A**) CircosPlot visualization of the RNA-Seq transcriptomic data regarding the genes predicted to encode phospholipases and esterases. *Zt105080*, *Zt109795* and *Zt81448* were shown to be highly transcribed in Δ*myco5* compared to WT and other the strains examined. (**B**) Imaging the lipid content of the Δ*myco5* strain. Localization of neutral lipids was visualized by Nile Red staining in IPO323 and the Δ*myco5* strain. BF, bright field microscopy; NR, Nile Red staining.

The RNA-Seq analysis of the Δ*myco5* strain indicated that the transcription of genes predicted to code for four lipases/lysophospholipases (*Zt41969*, *Zt74078*, *Zt107391* and *Zt109795*) and two triacylglycerol hydrolases (*Zt81448* and *Zt105080*) respectively were affected. Additionally, the triacylglycerol hydrolase–encoding genes (*Zt81448* and *Zt105080*) and a gene encoding a putative FabD lysophospholipase (*Zt109795*) showed increased transcript levels in Δ*myco5* mutant compared to the other strains examined. Transcriptome profiles are shown by using CircosPlot (Figure 9A). To establish if the differences seen in gene transcription for these genes encoding potentially lipid active enzymes could affect the lipid distribution or content in the Δ*myco5* strain, we used the Nile Red (9-diethylamino-5H-benzo[alpha]phenoxazine-5-one) stain, a vital stain established as selective for the detection of intracellular neutral lipid droplets, using fluorescence microscopy. We found a better selectivity for cytoplasmic lipid drops when the cells were examined at an excitation of 450–500 nm; emission, >528 nm rather than at excitation, 515–560 nm; and emission, >590 nm, as used in previous studies [44]. Employing this histochemical approach, the intracellular location and abundance of lipid bodies could be observed. Following three days’ growth either in N-deprivation or YEG liquid media, the Δ*myco5* strain showed fewer intracellular neutral lipid bodies than the IPO323 (WT) (Figure 9B). IPO323 grew predominantly yeast-like under the nutrient-rich condition (YEG), as we had anticipated, and these cells showed numerous small intense yellow bodies in conidia which had not germinated and were clearly discerned against a background of diffuse bright yellow fluorescence. In terms of growth under the same conditions, the Δ*myco5* mutant showed strongly enhanced filamentation and rarely exhibited yeast-like growth, and these hyphae fluoresced with a significantly weaker signal than WT. Notably, the small fraction of yeast-like cells of the Δ*myco5* mutant were more intensely fluorescent than the hyphae, and similarly to the wild type, bright yellow vesicles were observed. Moreover, staining intensity was less prominent in difference between WT and Δ*myco5* under N-deprivation (the dimorphic-switch-inducing situation). Both strains showed disperse yellow signals reduced in intensity, suggesting enhanced turnover of neutral lipids (data not shown). This finding indicates that the expression of the genes involved in lipid metabolism may be tightly regulated according to the nutritional state or growth mode.

### 2.10. The Δmyco5 Mutant Exhibits Reduced Proteolytic Activity

**Figure 10:**
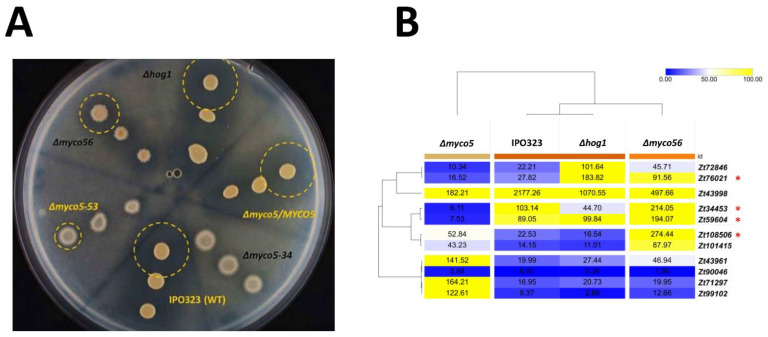
Δ*myco5* mutant shows reduced proteolytic activity. (**A**) Growth assay of the mutant strains and IPO323 on N-deprivation medium supplemented with 1% skimmed milk to visualize the proteolytic activity. Strains were inoculated as 1.5 µL drops of a 5 × 10^7^ spores/mL spore suspension and grown for 7 days at 18 °C. The clearing zones around the colonies show extracellular protease activity. Scale bar is 20 μm. (**B**) A heat map indicating the transcript abundance (FPKM values) of differentially expressed genes which are predicted to encode proteases. Transcript data were clustered in two dimensions by hierarchical agglomerative clustering with complete linkage. One minus Pearson correlation was employed as the similarity metric. Red asterisks (*) show DE genes which are predicted to code for extracellular proteases.

The RNA-Seq analysis that was performed revealed a set of DE genes encoding proteases when comparing the transcriptomes of Δ*myco5* and IPO323 strains. According to SignalP-analysis, the genes *Zt76021*, *Zt34453* and *Zt59604*, which are predicted to encode secreted proteases, exhibited decreased transcript levels in Δ*myco5*, suggesting an altered proteolytic activity of the mutant (Figure 10B). To examine extracellular proteases and the protease activity of the Δ*myco5* mutant, strains were grown on N-deprivation medium augmented with 1% skimmed milk as the only protein source. IPO323 and Δ*Zthog1* showed large clearing zones (halos), indicating proteolytic activity (Figure 10A). In contrast, the Δ*myco5* strain was markedly reduced in its ability to degrade skimmed milk. Importantly, the complemented strain Δ*myco5*/*MYCO5* had protease activity resembling the WT control strain, proving that this defect in proteolytic activity is indeed caused by the Δ*myco5* mutation.

### 2.11. Altered Cell-Wall Composition in the Δmyco5 and Δmyco56 Mutants

**Figure 11:**
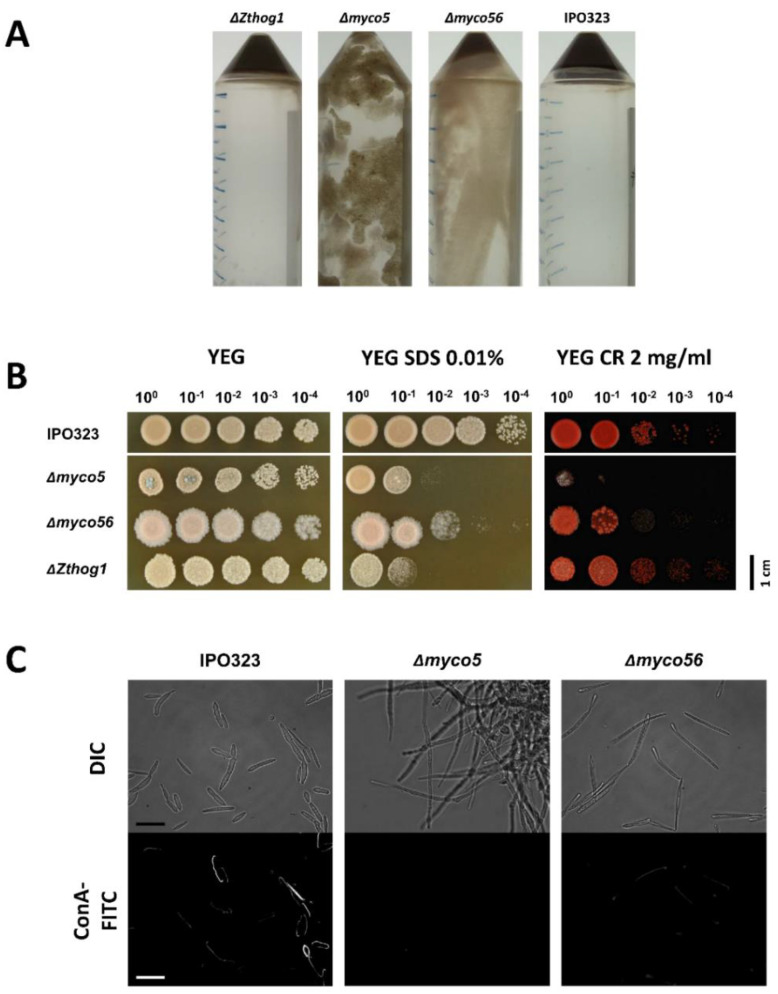
Involvement of *MYCO5* and *MYCO56* in cell-wall-integrity maintenance. (**A**) Pellet stability analysis was carried out by centrifugation at 4000 rpm of the strains cultured in YEG fluid medium for 4 days at 18 °C. After centrifugation, all pellets were directly documented. (**B**) Sensitivity of the mutant strains to cell-wall-perturbing agents. strains were grown on YEG medium with 0.01% SDS or 2 mg/mL Congo Red (CR) for 7 days at 18 °C. Four spots of 1.5 microliter of 1 × 10^8^ spores/mL spore suspension were inoculated on the plate. (**C**) Investigation of mutant strain’s cell wall toward the Concanavalin-A (ConA-FITC), using fluorescent microscopy. Δ*myco5* and Δ*myco56* showed altered staining compared to IPO323 both in the number of cells which bound ConA-FITC and the intensity of fluorescent signal. Scale bar = 20 μm.

Following centrifugation of the Δ*myco5* and Δ*myco56* mutants grown in YEG medium as submerged cultures for 4 days at 18 °C, pellets were noticeably more viscous in consistency compared to WT and Δ*Zthog1* pellets (Figure 11A). To test for cell-wall defects, the strains Δ*myco5* and Δ*myco56* were tested for hypersensitivity to cell-wall stressors, including the cell-wall polymer intercalating agent Congo Red (CR) and the lectin Concanavalin A-FITC (ConA-FITC). CR is considered to disrupt cell-wall synthesis by intercalating with chitin within the fungal cell wall. These tests revealed significantly reduced staining of Δ*myco5* and Δ*myco56* cell walls by ConA (Figure 11C), which is known to have a strong binding affinity for mannosyl-residues [45]. Although the application of 250 µg/mL Congo Red had no significant effect on any strain tested (data not shown), 2 mg/mL Congo Red drastically impaired the growth of the Δ*myco5* mutant (Figure 11B). Δ*myco56* also exhibited CR sensitivity at this concentration, because, although not as extremely affected as Δ*myco5,* the Δ*myco56* colony density was also reduced in comparison to IPO323. Additionally, growth in the presence of sodium dodecyl sulfate (SDS) was tested. SDS is a detergent considered to compromise cell-membrane integrity. Δ*myco5* and Δ*Zthog1* growth on YEG augmented with 0.01% SDS showed hypersensitivity compared to IPO323. Growth of Δ*myco56* under the same growth condition is reduced, indicating an alteration in cell-wall integrity. All cell-wall deficiencies could be restored in complemented mutants (data not shown).

## 3. Discussion

Dimorphism is considered a virulence/pathogenicity determinant with relevance not only in agriculture but also in medical mycology [8,9,10]. With the increasing prevalence of fungal diseases caused by dimorphic phytopathogenic and systemic dimorphic fungi, an understanding of the mechanisms controlling this morphogenic switch is of extreme biological, economical and therapeutical importance. The aim of the current work was to characterize two of the isolated random mutant strains defective in the dimorphic switch and to unveil the biological role of the corresponding genes for the dimorphic-switch process in *Z. tritici*. As expected, mutants that are defective in either gene were found to be non-pathogenic or at least exhibiting reduced virulence, since no symptoms of successful colonization of wheat were ostensible.

### 3.1. MYCO5 Negatively Influences the Dimorphic Switch and Is Involved in Diverse Biological Processes

*MYCO5* product appears to act directly or indirectly as a negative regulator of dimorphic transition, since inactivation of the gene results in mutants displaying enhanced mycelium formation under different incubation conditions, even on YEG medium, which normally maintains the yeast-like propagation of wild-type conidia. Many of phenotypes observed, including attenuated proteolytic activity, enhanced susceptibility to hydrogen peroxide, increased sensitivity to transient metals and enhanced susceptibility to cell-wall-perturbing agents, fit well with the transcriptional changes observed in the mutant, using RNA-Seq. Defining the nature of this regulation in more detail will be key in future studies. It will also be critical to determine what factors block the pathogenic development of this mutant strain, since it is proficient in filamentous growth, the morphogenic state that is considered to be required for successful host penetration. In this regard, it will be necessary to microscopically follow the pathogenic development in the mutant’s association with the wheat host. In general, strains exhibiting reduced virulence may be either deficient in host penetration or growth in planta. Inability to proliferate in planta might also be due to an inability to acquire essential nutrients or an inability to suppress host defense mechanisms [46]. Penetration defects might be due to reduced polarized growth, leading to a loss of entry into the host, as reported for the ZtFus3 MAP kinase [47]. Because the *MYCO5*-deleted mutant is proficient in filamentous growth, it is necessary to further investigate the proliferation of the mutant in planta. The fact that the mutant has increased susceptibility to hydrogen peroxide on artificial media is a strong first hint that suppression of the hypersensitive reaction may be deficient in the mutant. A susceptibility to the plant’s oxidative burst would also make sense when considering the differentially expressed genes in RNA-Seq analysis. It will be important to monitor the progression of the mutant in planta microscopically and to test whether we can see increased amounts of oxidative compounds within infections with the mutant by using stains such as DAB (3,3′-Diaminobenzidine), or whether infection can be restored by application of antioxidants. It will also be worth testing whether the mutant is compromised in the ability to detoxify plant-derived antimicrobial compounds, such as glycosylated triterpenoids (saponins), steroids or steroidal alkaloids. If a sensitivity to plant-defense metabolites can be established, this might be due to the MFS and MDR transporter genes with altered transcription in the mutant that we observed in the transcriptome analysis. The virulence-related role of similar transporters in interaction with hosts has been documented in other plant-pathogen fungi. For example, *M. oryzae ABC1* codes for an ATP-driven efflux pump and is purported to be an important component of the mechanism by which rice blast fungus can detoxify rice defense compounds, including phytoalexins [48].

Another question which should be addressed in a future study is exactly which metabolic pathway of lipid catabolism is responsible for the phenotypic alterations seen. We can envision both a role in energy supply via mitochondrial/peroxisomal β-oxidation of fatty acids and/or an impact on the remodeling of the membranes or additionally in lipid-based cell signaling. The DE gene products include predicted (lyso)phospholipases, esterases, epoxide hydrolases and glycosyltransferases, with conceivably varied functions, including membrane dynamics, signaling and cell-wall remodeling processes [49]. The activation of the β-oxidation pathway is supported by RNA-Seq data. The Acyl-CoA synthase encoding gene (*Zt39405*), being involved in fatty acid biosynthesis, exhibited significantly decreased transcript level in Δ*myco5*, whereas the Acyl-CoA dehydrogenase gene (*Zt38075*), which is involved in the first step of β-oxidation, showed increased transcript abundance in the mutant compared to other strains. The Nile Red fat distribution analysis revealed modest differences between in Δ*myco5* and IPO323 under conditions inducing the dimorphic switch (N-deprivation; data not shown). This suggests that the lipid catabolic enzymes seen among the predicted products of the DE genes may not be directly controlled by the *MYCO5* gene product, but perhaps these are coupled to the filamentous growth pattern as a secondary effect via the activity of signaling pathways. Additionally, the data are consistent too with a view that fats represent an energy source for the initiation of hyphal development and vegetative growth of the mycelium [7]. Still, a more detailed histological analysis of lipid distribution and a time course of relevant gene transcription, including, importantly, for both in planta, is required to fully understand how the observed alterations to the transcriptome impact the pathogenic development.

### 3.2. MYCO56 Encodes RNA-Binding Protein with a Role in Cell Branching, the Dimorphic Switch and Melanin Biosynthesis

*MYCO56* gene deletion led to pleiotropic effects on *Z. tritici* that include a distinctive conidial morphology with an elongated shape and reduced lateral conidial branching in the Δ*myco56* strain and an unusual pseudohyphal mode of growth. The dimorphic switch ability to hyphal growth was, however, drastically impaired, and the mutant strain is non-pathogenic. Similar phenotypes were not a feature of the *CIP2*-deficient *S. pombe* strains, thus implying the specific role of Myco56p in physiology and morphological processes in *Z. tritici*. A further functional discrepancy between *Z. tritici* Myco56p and *S. pombe* Cip2p is that *S. pombe CIP2* deletants show enhanced hydrogen peroxide tolerance, an effect not seen in Δ*myco56* mutants.

*MYCO56* deletion increased the pigmentation of colonies of the mutant, which became dark on N-deprivation medium. This phenotype makes sense when considered together with the RNA-Seq data. Transcriptome analysis showed that transcripts potentially coding for enzymes with a role in DHN-melanin biosynthesis showed increased abundance in Δ*myco56*. Melanin has been suggested to act as a virulence factor in many pathogenic fungi, including the phytopathogenic fungi *Magnaporthe oryzae* and *Verticillium dahlia* [50,51], as well as in several human pathogenic fungi, including *Paracoccidioides brasiliensis* and *Histoplasma capsulatum* [52]. Previous analyses have shown that melanin is required for successful plant penetration in *M. oryzae* [53]. Therefore, strains deficient in melanin biosynthesis are non-pathogenic in this fungus. In contrast, the role of melanin in *Z. tritici* remains unclear. Although *Z. tritici* does not penetrate plants directly as the rice blast fungus, potentially melanin has a role in *Z. tritici* for protection against toxic substances, and it will be important to test this in future studies.

### 3.3. Transcriptome Analysis Provides Crucial Insights into the Regulation of the Dimorphic Switch

To determine the transcriptional profile associated with the dimorphic switch and understand which genes are transcriptionally dependent on Myco5p, Myco56p and ZtHog1p during the yeast-to-hyphal transition, RNA-Seq analysis of IPO323 and the respective mutants was undertaken.

The RNA-Seq dataset revealed many DE genes having products with a diverse range of functions, including cell-wall biogenesis and organization, transporters and components of signal transduction pathways. These are very promising candidates for being key to the differentiation and proliferation associated with the dimorphic switch, and confirmation of this will be important in future research. For all the strains, we could document strain-specific gene sets, and, importantly, the inactivation of *MYCO56*, *MYCO5* and *HOG1* was confirmed in that no transcripts were found in the respective gene-deleted mutants (Supplementary Materials Figure S8). The gene transcription profiles highlighted major similarities and differences in the strains examined and may guide identification of factors associated with their strain specific patterns of yeast-like or filamentous growth. By Using PHI-Base, we identified DE genes whose products act as pathogenicity factors in other species. Of these genes, several were previously demonstrated to play a role in infectious development in other species, including in dimorphic fungi. Moreover, we found *Z. tritici*–unique genes differentially transcribed at the dimorphic switch and with no homologues or with only one, two or three homologues in other fungal genomes. Of these, many showed radically different strain-specific transcription profiles, signifying their potential role in dimorphism. Moreover, the product of these could prove to be promising candidates as fungicide targets for novel methods of crop protection. The DE genes (Supplementary Materials Table S2), together with the enrichment analysis (Figure 4) and PHI-Base interrogation results, highlight potential dimorphism-related processes that are worthy of further investigation and provide a basis for accelerating research on this economically important fungus. It will certainly be exciting to see which of the factors we have uncovered is crucial for the dimorphic transition and whether the findings here have broader significance in understanding dimorphic fungi more widely.

## 4. Conclusions and Outlook

In summary, the current study provided a preliminary functional analysis of two genes designated *MYCO5* and *MYCO56*, demonstrating their importance for dimorphic switch and pathogenicity in *Zymoseptoria tritici*. Phenotyping of the mutants generated in the study revealed several intriguing effects caused by respective gene inactivation and was guided by RNA-Seq data obtained, allowing us to show the transcriptional changes observed had real and expected phenotypic consequences for the fungus in line with the genes affected. The study provides several promising hints to the diverse biological processes which contribute to the regulation of the dimorphic switch of *Z. tritici*. Although our observations showed that *MYCO5* and *MYCO56* play an important role in the dimorphic switch of the *Z. tritici*, the precise molecular mechanisms underlying this phenotypic observation will require further investigation. This will ultimately lead to the discovery of further novel pathogenicity factors regulating the dimorphic switch and the wider pathogenic development of *Z. tritici* and its interaction with its wheat host.

Given the phenotypes of the mutants obtained, we aimed to examine the transcriptomes of the target mutant strains under nutrient-deprivation condition (N-deprivation). This allowed us to gain first insights into the dimorphism associated processes in *Z. tritici*. The RNA-Seq data provide a catalogue of genes that are potentially connected to the regulation of the dimorphic switch. Among these genes were some whose products participate in infectious development in other fungi that cause diseases of plants or humans. In addition, the bioinformatic analyses we employed revealed several previously undescribed DE genes which may have a unique role in dimorphic switch in *Z. tritici* and therefore might potentially be used as the basis for highly selective control measures. In all, by exploiting forward and reverse genetics, together with RNA-Seq transcriptomics, we have a powerful pipeline to identify candidate pathogenicity-related genes. This study lays the groundwork for further mechanistic investigations to expand our knowledge of the molecular mechanisms controlling the dimorphic switch. In conclusion, the genes we have pinpointed are promising candidates for being key factors for the control of the dimorphic switch in *Z. tritici*, and it will be of great interest to further clarify their exact biological roles.

## 5. Material and Methods

### 5.1. Strains, Growth Conditions and Oligonucleotides

The mutants in this work were derived from the wild-type *Zymoseptoria tritici* strain IPO323 (CBS Fungal Collection, Utrecht, NL, USA). IPO323 and the mutants generated were grown at 18 °C and 120 rpm in liquid YEG medium (Dextrose 10 g/L, yeast extract 10 g/L, pH 6.5) or cultivated at 18 °C on YEG-agar medium (2%). For phenotyping and growth assays using stressors, nitrogen-starvation medium (N-deprivation), minimal medium (MM), YEG and potato dextrose agar (PDA) media were employed. Nitrogen-starvation medium (pH 6.5), which represented the switch-inducing medium, and MM were prepared as described previously [54]. PDA was obtained from Carl Roth (Karlsruhe, Germany).

The oligonucleotides used are listed in the Supplementary Materials S1 File and were purchased from Eurofins-MWG-Operon (Ebersberg, Germany). All chemicals were sourced from Sigma-Aldrich (Munich, Germany), unless stated otherwise.

### 5.2. Nucleic Acid Manipulations

DNA manipulations and cloning procedures followed standard protocols [55]. All restriction endonucleases and the enzyme T4-DNA ligase were sourced from NEB. PCR for cloning purposes was used, Phusion High Fidelity Polymerase (New England Biolabs (NEB), Herts, UK). Diagnostic colony amplifications were made by using SapphireAmp^®^ Fast PCR Master Mix (Takara Bio, Inc., Otsu, Japan). All other PCR amplifications were made by using DreamTaq™ DNA Polymerase (Thermo Scientific, Schwerte, Germany), according to the recommendations of manufacturer. Plasmids were extracted from *E. coli* with the QIAprep spin mini-prep kit (Qiagen, Hilden, Germany). Isolation of *Z. tritici* genomic DNA routinely used the Qiagen DNeasy Kit (Qiagen, Hilden, Germany). The construction of vectors for gene deletion used the Gibson Assembly^®^ approach. Primers were designed by using the NEBuilder™ tool provided by NEB. Detailed strategies, as well as descriptions for specific transformation constructs, are given in the Supplementary Materials S2 File. To build the binary vectors used for targeted gene deletion, pCAMB0380 was used.

### 5.3. Pathogenicity Assays

For virulence test assays, conidia of the appropriate strains were harvested by centrifugation from 4-day-old liquid YEG cultures. Conidia were washed with DI-water and then adjusted to 1 × 10^7^ conidia/mL in 0.2% gelatin. Ten-day old seedlings of the wheat *cv. Riband* were used for infection assays, using the spray-inoculation method. Inoculation, growth and inspection of plants were carried out as previously described [54].

### 5.4. Multiple Alignment and Phylogenetic Study of Zymoseptoria tritici Amino Acid Sequences

DNA-sequence analyses, protein-sequence alignments and phylogenetic analyses were performed by using the software Geneious R11 11.1.5 [56]. All fungal protein sequences were retrieved from the Ensemble Fungi and JGI databases. We included *MYCO5* (*Mycgr3_66947*) and *MYCO56* (*Mycgr3_110503*) protein sequences and their homologues from the sister and closely related species *Z. pseudotritici* and *Z. ardabiliae*. In addition, we also included protein sequences of the genes with sequence similarity to Myco5p and Myco56p based on the next best BlastP search hits, which were used as outgroup for tree construction. The alignment of protein sequences was performed by using MUSCLE [57]. The phylogenetic trees were constructed by using the “neighbor-joining”-algorithm based on the Jukes–Cantor genetic distance model. The statistical accuracy of the consensus tree was tested by bootstrap analysis, including 105 replicates.

### 5.5. RNA-Seq Analysis

To investigate the transcriptomic profiles connected to the dimorphic switch, the Δ*myco5* and Δ*myco56 ZtHOG1* mutants and the wild-type strain IPO323 were used. For RNA isolation, 300 µL of the spore suspension of these strains with a final concentration of 10^6^ spores/mL was plated on N-deprivation-agar plates overlaid with sterile cellophane and grown for 7 days at 18 °C to obtain synchronous cultures. Total RNA extraction was made with the RNeasy Plant Mini Kit (Qiagen Sciences, Valencia, CA, USA), according to the manufacturer’s instructions. Before RNA extraction, samples (cellophane plus fungal biomass) were lyophilized and then ground to a fine powder, using liquid nitrogen. The quality and integrity of the RNA isolated was assessed by using the Bioanalyzer 2100 (Agilent, Waldbronn, Germany). All RNA had a 260 nm/280 nm ratio in the range of 1.8–2.1 and a 28S/18S ratio within 1.5–2.0. RNA concentration was evaluated by using an ND-1000 NanoDrop spectrophotometer (NanoDrop Technologies, Wilmington, DE, USA). Library preparation was performed by GENterprise Genomics (Mainz, Germany) with 1.5 μg total RNA for each sample and sequenced in a 150 bp paired-end run on an Illumina HiSeq 2500 (IMSB, Mainz, Germany). Data processing for transcriptome assembly and differential transcription analysis used the RNA-Seq processing pipeline based on TopHat-Cufflinks-Cuffdiff [58]. Raw paired-end RNA-Seq reads were quality checked with FastQC (http://www.bioinformatics.babraham.ac.uk/projects/fastqc/; last accessed on 1 January 2019), trimmed if required and then were merged with the aid of FLASH 1.2.9. Merged paired reads were then aligned to the IPO323 reference genome with the help of the program TopHat v1.3.3 [59], using the standard options. As input parameters, the genome builder Zymoseptoria_tritici.MG2.30.dna.genome.fa.gz in EnsemblGenomes (ftp://ftp.ensemblgenomes.org/pub/fungi/release-30/fasta/zymoseptoria_tritici/dna/; last accessed on 1 December 2021) and the annotation file Mgraminicolav2.FilteredModels1.gff, which is based on filtered gene models at the JGI server (http://genomeportal.jgi-psf.org/Mycgr3/Mycgr3.home.html; last accessed on 1 December 2021), were used. The fragment length parameter was set at 200 bp, with a standard deviation of 100 bp. The Integrative Genomics Viewer (IGV) [60,61] allowed for the visualization of the assembled transcripts. Cuffdiff was then used to calculate expression levels (FPKM values) for the annotated genes on the reference genome. To search the processed data and to envision the differential expression patterns, CummeRBund 2.0 analysis and the visualization package for Cufflinks high-throughput sequencing data in R/Bioconductor was used. The transcript abundance for the genes was reported as normalized fragments per kb of transcript per million mapped reads. Transcripts with a significant *p*-value (≤0.05) and a greater than 1.5 (log2)-fold change in transcript abundance were noted as differentially expressed, unless stated otherwise. All the *p*-values were adjusted for false discoveries arising from multiple hypothesis testing, using the Benjamini–Hochberg method.

### 5.6. qRT-PCR Analysis of (Chloro) Peroxidases and Dismutases Encoding Genes

To assess the transcription of the predicted (chloro)peroxidases-encoding genes in the infection cycle, infected plant leaves were sampled at varied time points, up to 28 dpi. RNA isolation was undertaken by using the RNeasy Plant Mini Kit (Qiagen Sciences, Valencia, CA, USA), and the relative transcription of the genes was monitored by qRT-PCR, using the iQ SYBR Green Supermix Kit (Bio-Rad, Munich, Germany) with gene-specific primers and by annealing at 59 °C, with 1 dpi as a reference. The PCR was carried out by using a CFX96 Real-Time PCR Detection System (Bio-Rad). Transcript levels were determined by using two biological replicates with three technical replicates, following the Livak method, a modification of the original Pfaffl method [62,63]. The housekeeping gene encoding β-tubulin (*Zt102950*) was used as a constitutively transcribed control (house-keeping gene). Comparisons of the relative transcript abundance of the target genes used the average Ct value normalized to that of β-tubulin for each sample as 2^−ΔCt^, where 2^−ΔCt^ = (Ct_(target gene)_ − Ct_(β-tubulin)_). Oligonucleotides sequences employed in qPCR analysis are given in Supplementary Materials File S1.

### 5.7. RNA-Seq Data Analysis of the Transcription of Known Pathogenicity Related Genes

The predicted amino acid sequences of the DE genes were interrogated with recognized pathogenicity-related proteins from different fungal pathogens. For this, a custom database was created containing the established virulence determinants from the pathogen–host interaction (PHI) database [64]. This interrogation using the custom database was carried out by using local BLASTp with a minimum cutoff value of ≤10^−30^. The sequences identified were then reanalyzed to remove any false positives from the dataset.

### 5.8. Additional Analyses/Gene Ontology Analysis

Gene ontology and gene enrichment analysis of the differentially transcribed genes identified by using RNA-Seq was carried out with the aid of Blast2GO Pro, TopGO and REVIGO [65,66,67]. The Blast2GO analysis tool for functional annotation worked by recovering descriptions of the predicted gene product’s function, using the standardized vocabulary of the gene ontology (GO) bioinformatics initiative. The output of the BlastGO enquiry was used to refine and update the GO annotation sourced from JGI. The GO terms resulting were then additionally processed, using TopGO analysis and Fisher’s Exact Test to obtain an inventory of significantly enriched GO clusters, using a corrected *p*-value of 0.05. The top 50 GO clusters for each of the strains investigated were then used for REVIGO analysis (http://revigo.irb.hr, last accessed on 1 December 2021) by discovering over-represented GO categories of the differentially transcribed genes (*p* < 0.05) and thus giving a global depiction of biological processes affected. This was accomplished by grouping related terms based on their semantic similarity and then by removing existing redundancies. Default parameters were employed, and the redundancy threshold (allowed similarity) was set at 0.7 (medium). The output was the enriched GO clusters, specific for each of the strains, which were then visualized as tree maps, with the assistance of the R package *ggplot*.

## Data Availability

The data generated for this study are available upon request to the corresponding author.

## Supplementary Materials

The following are available online at https://www.mdpi.com/article/10.3390/ijms23010400/s1.
