# Supplementary material for: Two Novel Dimorphism-Related Virulence Factors of Zymoseptoria tritici Identified Using Agrobacterium-Mediated Insertional Mutagenesis
Source: Int J Mol Sci. 2021 Dec 30;23(1):400. doi: 10.3390/ijms23010400 (PMC8745584; doi:10.3390/ijms23010400)
Supplement: Supplementary file 1 [file ijms-23-00400-s001.zip › S1_File.pdf]

Summary of primers used in this work

Primers used for generation of gene inactivation constructs. Lower case sequences represent the tails of the primers used for overlapping of fragments within Gibson assembly reaction.

Primer name	Direction	Sequence (5‘ → 3‘)
**myco5-fr.1-for**	sense	ggcccggcgcgccgaattcccggggatccgTTGGCTTCTTTAGCTGCGGC
**myco5-fr.1-rev**	antisense	ttcaatatcatcttcGATAAGACGAAGACCAATCTGACG
**myco5-fr.2-for**	sense	ggtcttcgtcttatcGAAGATGATATTGAAGGAGCATTTTTG
**myco5-fr.2-rev**	antisense	ggtaggggatggatcTCTTGTTCGGTCGGCATCTA
**myco5-fr.3-for**	sense	gccgaccgaacaagaGATCCATCCCCTACCGCACTT
**myco5-fr.3-rev**	antisense	gtcagatctaccatggtggactcctcttaaACTGTCGATGCAGCGACGAT
**myco56-for**	sense	CTCGATGCTTGCGCCGGTCT
**myco56-rev**	antisense	TCCGGACGAGTGGCGATGGT

Primers used for complementation constructs

Primer name	Direction	Sequence (5‘ → 3‘)
**myco5-Comp-for**	sense	GCCTTTTCCTTGGCCTCCTTCAA
**myco5-Comp-rev**	antisense	CCGACTGATTGCCCACGGGA
**myco56-Comp-for**	sense	GCGAGAGGCCGAGCAAGTGT
**myco56-Comp-rev**	antisense	TGCGGCGGCATCGATACGTG

Primers used for Screen PCR

Primer name	Direction	Sequence (5‘ → 3‘)
**myco5-fr.1-for**	sense	ggcccggcgcgccgaattcccggggatccgTTGGCTTCTTTAGCTGCGGC
**myco5-fr.3-rev**	antisense	gtcagatctaccatggtggactcctcttaaACTGTCGATGCAGCGACGAT
**myco56-for**	sense	CTCGATGCTTGCGCCGGTCT
**myco56-rev**	antisense	TCCGGACGAGTGGCGATGGT

Primers used for probe generation (for Southern Blot analysis)

Primer name	Direction	Sequence (5‘ → 3‘)
**myco5-probe-for**	sense	CTGAAGGTACTGCGCTGTCT
**myco5-probe-rev**	antisense	CAGGGCTCTAGAGATGCCTC
**myco56-probe-for**	sense	GCCGTCCGCATCGCAAAAGG
**myco56-probe-rev**	antisense	AACGCAGGATTCGCCGGTGG

Primers used for qRT-PCR

Primer name	Direction	Sequence (5‘ → 3‘)
**myco-b-tub-for**	sense	CTTCCGCCCAGACAACTTCGT
**myco-b-tub-rev**	antisense	TCTGGAAACCCTGGAGGCAGT
**Zt43487-qPCR-for**	sense	CGCTCTTTTCGAGGCCATTC
**Zt43487-qPCR-rev**	antisense	AGCCATTTCGGGGTAAGAGG
**Zt74298-qPCR-for**	sense	TGGACAACTCCATGCTCTCC
**Zt74298-qPCR-rev**	antisense	TCCACCTTTTCTGGCGTTGT
**Zt94368-qPCR-for**	sense	CGATCTTCGAGGCCGTTCAA
**Zt94368-qPCR-rev**	antisense	GCTGCCCTCTGAAATGTCGAT
**Zt96677-qPCR-for**	sense	GACAAGGAGGCGCAGTACAT
**Zt96677-qPCR-rev**	antisense	CTGCTGGATGGTGGCGTAT
**Zt101235-qPCR-for**	sense	CCGCAGCAAACCACAACTTC
**Zt101235-qPCR-rev**	antisense	TGACTAGATCGCCATCGGTG
**Zt102956-qPCR-for**	sense	AGAATGCGCTCGGAGTCAC
**Zt102956-qPCR-rev**	antisense	GAGCAGTTGCCGTTGGTGA
