# Supplementary material for: Two Novel Dimorphism-Related Virulence Factors of Zymoseptoria tritici Identified Using Agrobacterium-Mediated Insertional Mutagenesis
Source: Int J Mol Sci. 2021 Dec 30;23(1):400. doi: 10.3390/ijms23010400 (PMC8745584; doi:10.3390/ijms23010400)
Supplement: Supplementary file 1 [file ijms-23-00400-s001.zip › S2_File.pdf]

Plasmid construction for targeted inactivation and complementation of the target genes in *Zymoseptoria tritici*

<u>*MYCO5*</u> (Zt66947; XP_003857106.1; Mycgr3_66947; JGI Protein ID: 66947):

For deletion of *MYCO5* gene the generation of the gene deletion construct was conducted using the Gibson Assembly® approach. Therefore, three required fragments were amplified by PCR using the primer pairs containing the overhangs overlapping with the neighboring fragments. The primer pairs myco5-neu-fr.1-for and myco5-neu-fr.1-rev as well as myco5-neu-fr.3-for and myco5-neu-fr.3-rev were used to amplify the fragments representing the flanking regions of the myco5 gene using genomic DNA as template. For the amplification of *HPT* cassette from the pCB1636 the primers myco5-neu-fr.2-for and myco5-neu-fr.2-rev were used. Thereby it was possible to generate the desired final deletion vector pCAMB-myco5-HPT-Final within a single assembly reaction. The generated transformation plasmid was verified by restriction analysis and could be directly used for ATMT.

For complementation of the *MYCO5*-deleted mutant the construct pCAMB-myco5-Comp-BAR was generated by amplification of the WT gene locus from strain IPO323 using the primers myco5-Comp-for and myco5-Comp-rev. The amplified PCR product was ligated to pGEM-T easy and then the insert excised using *Not*I and cloned into the vector pCAMB-BAR (7747 bp) restricted with *Psp*OMI. The generated final vector was verified by restriction analysis and could be directly used for transformation of hygromycin resistant mutants.

<u>*MYCO56*</u> (Zt110503; XP_003850341.1; Mycgr3_110503; JGI Protein ID: 110503):

For inactivation of *MYCO56* gene a 3657 bp PCR product was amplified using the primers myco56-for and myco56-for from genomic DNA of *Z.tritici* IPO323 and cloned into pJet (Promega, Mannheim; Germany) giving the resulting vector pJet-myco56KO. In the next step this vector was cleaved by *Sal*I and a 1962 bp fragment of the coding sequence was replaced by a *HPT* cassette from the *Sal*I restricted pCAMB-HPT(*Sal*I) to give pJET-myco56-HPT. Finally, the *Xba*I+*Not*I restricted fragment of pJET-myco56-HPT was cloned into *Spe*I+*Psp*OMI resctricted pCAMB0380 to give the completed gene inactivation vector pCAMB-myco56-HPT-Final. The generated transformation plasmid was verified by restriction analysis and could be directly used for ATMT.

For complementation of the *MYCO56*-disrupted mutant the construct pCAMB-myco56-Comp-BAR was generated by amplification of the WT gene locus from strain IPO323 using the primers myco56-Comp-for and myco56-Comp-rev. The amplified PCR product was ligated to pGEM-T easy to give pGEM-myco56-Comp. Subsequently the insert excised using *Not*I was cloned into the vector pCAMB-BAR (7747 bp) restricted with *Psp*OMI. The generated final vector pCAMB-myco56-Comp-BAR was verified by restriction analysis and could be directly used for transformation of hygromycin resistant mutants.
