# Supplementary material for: Two Novel Dimorphism-Related Virulence Factors of Zymoseptoria tritici Identified Using Agrobacterium-Mediated Insertional Mutagenesis
Source: Int J Mol Sci. 2021 Dec 30;23(1):400. doi: 10.3390/ijms23010400 (PMC8745584; doi:10.3390/ijms23010400)
Supplement: Supplementary file 1 [file ijms-23-00400-s001.zip › Supporting information.pdf]

Figure S1: **Phylogenetic comparisons of *Zymoseptoria tritici* (*Z.t.*) the *MYCO5* and *MYCO56* genes with the corresponding predicted fungal homologues based on amino acid sequence alignments.** The amino acid sequences are sourced from either the UniProt or JGI databases. Sequence alignments were carried using the program MUSCLE. Trees show phylogenetic relationships of **(A)** Myco5p and **(B)** Myco56p with homologues identified in the species *Aspergillus nidulans* FGSC A4 (*A.n.*), *Aspergillus fumigatus* Af293 *(A.f.)*, *Blastomyces dermatitidis* SLH14081 *(B.d.)*, *Candida albicans* SC5314 (*C.a.*), *Coccidioides immitis* RS (*C.i.*), *Colletotrichum graminicola* M1.001 (*C.g.*), *Fusarium graminearum* PH-1 (*F.g.*), *Histoplasma capsulatum* NAm1 *(H.c.*), *Neurospora crassa* ATCC 24698 (*N.c.*), *Pyricularia oryzae* 70-15 (*P.o.*), *Pseudocercospora emusae* CBS114824 (*P.e.*), *Paracoccidioides brasiliensis* Pb03 (*P.b.*), *Saccharomyces cerevisiae* S288C (*S.c.*), *Sphaerulina musiva* SO2202 (*S.m.*), *Penicillium marneffei* ATCC 18224 (*P.m.*), *Ustilago maydis* FGSC 9021 (*U.m.*), *Zymoseptoria ardabiliae* STIR04_1.1.1 *(Z.a.*), *Zymoseptoria brevis* Zb18110 *(Z.b.*) and *Zymoseptoria pseudotritici* STIR04_2.2.1 (*Z.p.*). The phylogenetic trees were created using "neighbour-joining"-algorithm based on the "Jones-Taylor-Thornton (JTT)" - model. The statistical accuracy was tested by bootstrap analysis with 100000 replicates. Support of nodes by percentage of bootstrapping iterations is indicated. Deduced: target gene *MYCO5* in *Z.p.* identified by BLASTp and Mauve analysis. The genes *MYCO5* and *MYCO56* are denoted by asterisk (*). The non-orthologues sequences of *Mycgr3_103744* and *Mycgr3_73484* formed an outgroup.

Figure S2: **Strategy employed for generation of the *Δmyco5* strain by targeted gene deletion and a Southern Blot showing successful gene replacement. (A)** Gene structure of *MYCO5* and the domain structure of its product found using the program InterProScan. The regions with predicted functional domains are highlighted in green. **(B)** Representation of the gene deletion performed with replacement of the majority of the ORF with *HPT* selectable marker. Location of sequence used for probe hybridization and the restriction enzyme sites used for digestion prior to Southern Blot analysis are shown. **(C)** Southern Blotting using gDNA from IPO323 and the candidate *Δmyco5* mutants cut with *Sac*I and following electrophoresis and transfer to a membrane probed with the 334 bp amplicon of the primers myco5-probe-for/myco5-probe-rev. **(D)** A PCR screen (using the primers myco5-fr.1-for and myco5-fr.3-rev) was further used as confirmation of the Southern blot results and indicates loss of *MYCO5* gene as well as a reinstated *MYCO5* copy in the complementation strain *Δmyco5*/*MYCO5*.

Figure S3: **The strategy employed to generate the *Δmyco56* strains using targeted gene deletion together with the Southern Blot providing evidence of gene inactivation. (A)** Gene structure of *MYCO56* and the domain structure in the gene product found using InterProScan. Functional domains are indicated in green. **(B)** representation of gene inactivation by removing the majority of the ORF and replacing with the *HPT* selectable marker. Position with the genomic sequence used for probe hybridization and restriction sites employed in Southern Blot analysis are indicated. **(C)** Southern Blot analysis. Genomic DNA from the wildtype strain IPO323 and the potential *Δmyco56* mutants was cut with *Xho*I and then probed with the 335 bp fragment amplified with the primers myco56-probe-for and myco56-probe-rev. **(D)** The PCR screen employed to indicate loss of the *MYCO56* gene in the suspected *Δmyco56* mutant and the presence of an additional gene copy in the complementation strain *Δmyco56*/*MYCO56*, with the aid of the primers myco56-for and myco56-rev.

<u>Figure S4:</u> Global statistics and quality control of RNA-Seq data. (A) Density plot showing the probability densities of transcription levels of genes in each of the strains used. **(B)** Boxplots showing gene distribution at each mean FPKM value across the strains used. These data established no reduction of values (reads) across the strains examined, evidencing that the sequencing depth and coverage were appropriate.

<u>Figure S5:</u> Identification of the most similar genes to a given target gene. Evaluation was carried out using the *findsimilar()*-function within in CummeRbund package in R. The similarity is supported using the Jensen-Shannon distance between the probability distributions of each of the genes across the strains used. The Top 20 most alike genes to a provided transcriptome profile of **(A)** *MYCO5* gene, **(B)** *MYCO56* gene (*Zt110503*) and **(C)** *ZtHOG1* gene (*Zt76502*) across strains used is shown. The target genes are marked by red asterisks.

<u>Figure S6:</u> *In planta* transcription pattern of the *Zymoseptoria tritici* chloroperoxidase encoding genes through infection. Fold changes show a relative quantification of the transcription levels of the selected genes at various stages of the infection course and relative to the transcription observed when grown in the nutrient replete condition (YEG); normalization was carried out with the constitutively expressed β-tubulin-encoding gene. Leaves of wheat cv. Riband inoculated with the wildtype strain IPO323 were taken 4, 10, 14 and 28 dpi. Error bars show the standard deviation of the three technical replicates. The experiment was performed twice, and similar results were obtained. The colored bars show the temporal transition from the biotrophic (green) to the necrotrophic (yellow, orange and red) phase.

<u>Figure S7:</u> Depiction of the transcription deleted gene loci in the mutant strains investigated and relative to that of the wildtype strain IPO323. Integrative Genomics Viewer (IGV) screen captures of the reads from RNA-Seq analysis mapped to the target gene loci in the respective mutants used. The blue boxes show gene models based on the JGI annotation while grey bars show sequencing reads. Blue lines connect the sequencing reads that are aligned over splice sites of adjoining exons. Red rectangles depict lack of sequencing reads for corresponding transcripts in mutant strains and validate successful deletion of the target genes.

<u>Figure S8:</u> Principal components analysis (PCA) of the gene transcription profiles of mutants used. PCA reduces the dimensionality of RNA-Seq data with the aid of linear combinations of gene transcription values to delineate a new set of unrelated variables (principal components). This allows clustering and visualization of the relationship between the gene profiles by their variance and description of datasets with reduced numbers of variables. These new variables are orthogonal and therefore avoid redundant information. Using this analysis, we found that *ΔZthog1*, *Δmyco56* and IPO323 form one cluster, indicating a similarity in their gene transcription profiles and are distinct from that of the *Δmyco5* Mutant ("varnames" used were: ipo323 for the wildtype reference strain IPO323, hog1 for the deletion mutant strain *ΔZthog1*, myco5 for mutant *Δmyco5* and myco56 for mutant *Δmyco56*).

<u>**Table S1:**</u> Comparative analysis RNA-Seq data with publicly available data from previous studies [**Error! Reference source not found.**-**Error! Reference source not found.**]. The most significantly up-regulated genes across the strains used are shown in green, with the down-regulated genes indicated in red.

Table S2: RNA-Seq analysis with the strains *Δmyco5*, *Δmyco56*, *ΔZthog1* and the wildtype strain IPO323 under nitrogen deprivation (following 7 days incubation). Shown are the Protein IDs from JGI, the FPKM values from CuffDiff analysis, the best match to characterized proteins (BLASTp, NCBI), the predicted function after Blast2GO analysis designated as the customized category, the SignalP 4.1 prediction results and PHI-Base comparison results. PHI-Base Pathogen Host Interactions database is an expertly curated molecular and biological database of genes known to affect the outcome of pathogen-host interactions (accessible via: http://www.phi-base.org/). PHI-base terms are depicted with: red "Loss of pathogenicity", orange "reduced virulence" and green "increased virulence or unaffected pathogenicity".

S1 File: Oligonucleotides used in the current study

S2 File: Strategy for construction of the vectors used for targeted deletion and complementation of the mutants in used in the current study
