# Supplementary figures and images for: Two Novel Dimorphism-Related Virulence Factors of Zymoseptoria tritici Identified Using Agrobacterium-Mediated Insertional Mutagenesis
Source: Int J Mol Sci. 2021 Dec 30;23(1):400. doi: 10.3390/ijms23010400 (PMC8745584; doi:10.3390/ijms23010400)

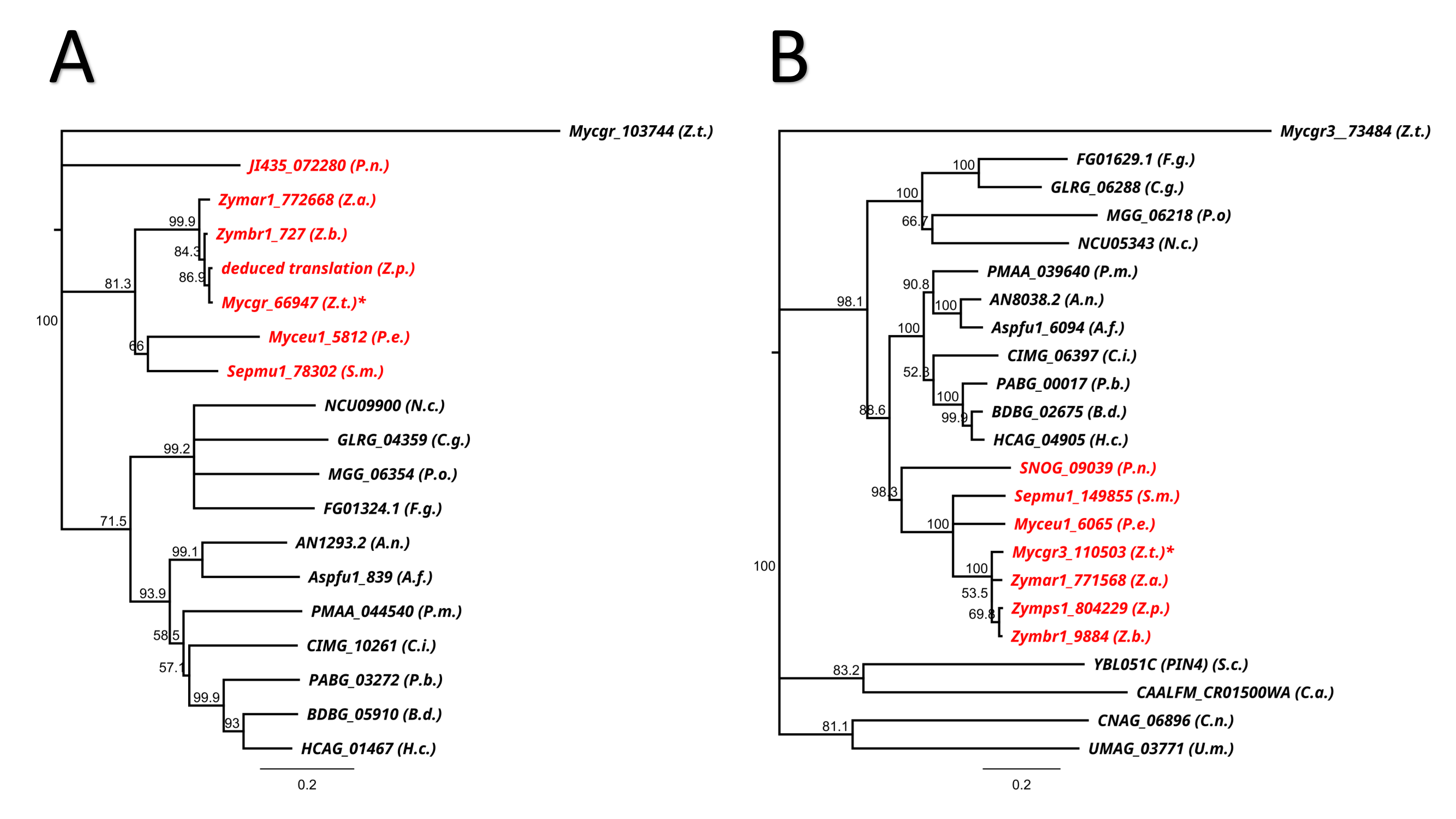

Supplement: Supplementary file 1 [file ijms-23-00400-s001.zip › S1_Fig.tif]
